# Improving Compliance With Getting It Right First Time (GIRFT) Appendectomy Documentation Guidelines: A Two-Cycle Audit at a District General Hospital in the United Kingdom

**DOI:** 10.7759/cureus.89833

**Published:** 2025-08-11

**Authors:** Lakshay Singla, Neeraj V Bagade

**Affiliations:** 1 General Surgery, Mersey and West Lancashire Teaching Hospitals NHS Trust, Prescot, GBR

**Keywords:** bsoap format, clinical data standards, clinical documentation, compliance improvement, girft (getting it right first time), laproscopic appendectomy, quality improvement projects, standardized templates

## Abstract

Good operative documentation is essential for clinical communication, quality assurance, and medicolegal protection. In the United Kingdom (UK), standardized documentation for laparoscopic appendectomies has been emphasized through national quality initiatives. We conducted a two-cycle audit at a UK district general hospital to evaluate compliance with 34 recommended standards for appendectomy documentation. The first cycle retrospectively included 75 cases from May to July 2024, while the second cycle prospectively included 37 cases from September to November 2024. Between the two cycles, interventions were implemented, including a structured operative note template and staff education. Post intervention, notable improvements were observed in documentation practices, particularly in the use of the electronic system (41.8% to 76%), mesoappendix documentation (78.1% to 100%), and reporting of blood loss (1.35% to 13.6%). The audit demonstrates that simple systematic changes can significantly enhance documentation quality. Ongoing use of structured templates and regular re-auditing are recommended to maintain high standards.

## Introduction

Laparoscopic appendectomy (LA) is among the most commonly performed emergency surgical procedures across the globe [1]. Effective documentation serves as proof of safe and high-quality care [2]. It enhances clarity and facilitates clear communication among the entire multidisciplinary team, helping them collaborate efficiently to provide well-coordinated, patient-centred care.

The Getting It Right First Time (GIRFT) programme is a nationwide initiative by NHS England aimed at enhancing patient care and treatment by conducting comprehensive service reviews, benchmarking performance, and using data-driven evidence to inform and support improvements [3]. The GIRFT model comprises five strands: (i) a national data analysis to review current practice and outcomes, (ii) direct clinical engagement with individual hospital trusts to compare local performance against the national picture, (iii) a national report highlighting opportunities for improvement, (iv) an implementation phase supporting trusts and systems to deliver changes, and (v) best practice guidance to standardise pathways and aid elective recovery in high-volume, low-complexity specialties.

Incomplete surgical operative notes can pose serious legal and ethical issues. From a legal standpoint, insufficient documentation may lead to questions about the quality of care delivered, increasing the risk of malpractice claims [4]. Ethically, poor record-keeping can jeopardize patient safety and disrupt continuity of care, as vital details necessary for effective postoperative management may be omitted. Precise and comprehensive operative notes are crucial not only for medicolegal safeguarding but also for maintaining high ethical standards in the delivery of patient care [5].

Electronic patient records (EPRs), also referred to as electronic health records (EHRs), are digital systems that capture and store a patient's healthcare interactions over time [6]. These systems compile comprehensive information such as clinical notes, diagnoses, medical history, and test results, ensuring easy access for NHS staff, researchers, and, increasingly, for the patients themselves. This audit aimed to evaluate adherence to 34 GIRFT documentation standards in laparoscopic appendectomy at our district general hospital and to assess the impact of implementing a structured electronic operative note template on documentation quality.

## Materials and methods

This two-cycle clinical audit was conducted in the Department of General Surgery at Whiston Hospital, a District General Hospital in Rainhill, United Kingdom, under the Mersey and West Lancashire Teaching Hospitals NHS Trust. The audit was registered with the hospital audit department (Audit IDs: GenSur/CA/2023-24/06 and GenSur/CA/2024-25/34) and did not require ethics approval. No patient-identifiable information was collected. The aim of the audit was to assess compliance with 34 documentation standards recommended for laparoscopic appendectomy procedures and to evaluate the impact of a structured electronic operative note template.

Cycle 1

Cycle 1 involved a retrospective review of the medical records of all patients who underwent laparoscopic appendectomy between May and July 2024. A total of 75 cases were included. Data were extracted from operative notes using a predefined proforma based on the 34 documentation standards (Table 1) [3]. These standards covered domains such as preoperative assessment (e.g., consent, imaging), intraoperative technique (e.g., use of ports, diathermy, blood loss), and postoperative elements (e.g., venous thromboembolism (VTE) prophylaxis, drain removal, follow-up).

**Table 1:** GIRFT operative documentation standards for laparoscopic appendectomy. The table summarises the 34 recommended documentation elements outlined by the “Getting It Right First Time” (GIRFT) national quality improvement programme in the United Kingdom [3]. These standards aim to ensure comprehensive operative note keeping, improve clinical communication, support quality assurance, and provide medicolegal protection.

Serial Number	Recommendation
1	The indications for the operation and the evidence, both in terms of serological markers, imaging, presenting complaint, and clinical examination that have led to the recommendation to perform this operation.
2	Record the time the decision was made to perform surgery, and also if surgery was subsequently deferred and the reasons.
3	Documentation of the informed consent process, including the risks of not operating, should be available. The likelihood of a blood transfusion or the need to proceed to an open procedure, a laparotomy, or any other additional procedures as relevant should be recorded along with the associated risks. It should be clearly documented if the patient does not consent to any of these relevant procedures, including transfusion.
4	Safety briefing, sign in, time out, and sign out as part of the WHO Surgical Safety Checklist. Presence of required surgical equipment for both laparoscopic and open procedures should be confirmed.
5	Record names of all surgeons with name/grade of lead surgeon and assistants.
6	Record names and grade of anaesthetist(s) and type(s) of anaesthetic used.
7	Record the date and time of the procedure.
8	Record drugs given pre-operatively and during surgery, e.g. antibiotics, local anaesthetic.
9	Record the insertion of a urinary catheter if carried out.
10	Record patient position and skin preparation.
11	Describe or draw the location of the incisions.
12	Record the open or closed technique used to enter the peritoneal cavity with a Hassan or Verres needle, and that the pneumoperitoneum was established prior to laparoscope insertion.
13	Confirm that the laparoscope was inserted into the abdomen under direct vision.
14	Document the insertion of other ports, their location, size, and whether local anaesthetic was used.
15	Record the level at which the intra-abdominal pressure was set.
16	Record if the patient was placed in the Trendelenburg position with the left side down.
17	Document the findings at the time of surgery, including appendix appearance and any associated pathology.
18	If the appendix appeared normal, document further actions taken to determine the diagnosis.
19	Record how the appendix was located and whether it was easily identified.
20	Record whether the appendix was ligated between endo-loops using laparoscopic scissors or stapled.
21	Record whether the appendiceal artery was simply divided by cautery or needed to be divided between clips.
22	Record whether the remainder of the mesoappendix was dissected and whether the base of the caecum was healthy and intact.
23	Record the volume of irrigation used for washout.
24	Document whether the appendix was placed in a sterile endoscopic bag for extraction.
25	Record whether the appendix was removed whole and if any residual tissue was left behind.
26	State that the tissue sample was sent to pathology.
27	Record that haemostasis was achieved.
28	Record that the ports were removed under direct vision.
29	Record any intraoperative complications, conversion to open, additional procedures, and rationale.
30	Record closure details, whether the fascia was closed, and if drains were used.
31	Document estimated blood loss.
32	Document the postoperative plan, including: antibiotics, blood tests, transfer location, frequency of observations, drain removal, pathology checks, VTE prophylaxis, postoperative recovery instructions, discharge plans, suture removal, and follow-up.
33	Record any images taken during the procedure and attach them to the operation record.
34	Signature of the first surgeon alongside their name and grade to confirm completeness and accuracy.

**Figure 1:**
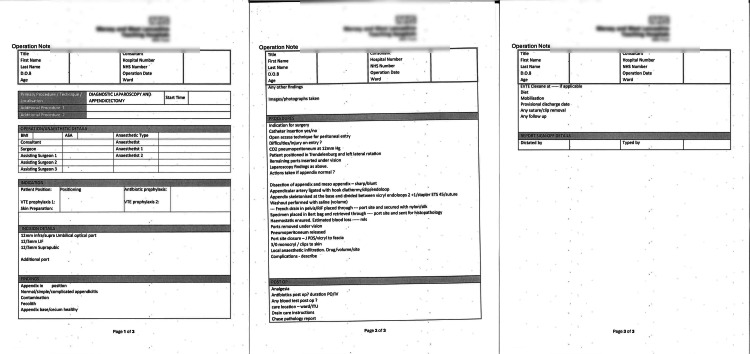
Operative notes template This was developed in accordance with Getting It Right First Time (GIRFT) recommendations. The template is designed to improve standardisation and completeness of operative documentation, ensuring compliance with national best practice guidelines. Panels from left to right depict sequential sections of the template.

Following Cycle 1, two key interventions were introduced: (i) Implementation of a structured operative note template integrated into the NHS OPERA electronic documentation system (Figure 1), and (ii) Educational sessions for surgical trainees and consultants to raise awareness of documentation standards. These were delivered via departmental meetings, emails, and targeted teaching.

Frequency, Duration, and Format

Two formal teaching sessions were conducted, each lasting 30 minutes, during the departmental audit supplemented by weekly email reminders over a four-week period. An additional 10-minute focused teaching was delivered at the start of each on-call shift during that period by the audit lead.

Content of Educational Intervention

The sessions covered the importance of documentation as per GIRFT recommendations, introduced a printed summary of GIRFT appendectomy documentation standards, and included a live demonstration of documentation via the OPERA system using anonymized examples.

Target Audience and Participation

The target audience included all surgical senior house officers (SHOs), registrars, and Foundation Year 2 (FY2) doctors rotating through general surgery. Attendance at the departmental sessions was approximately 85%, and all doctors received the distributed material via email and physical handouts.

Feedback and Comprehension

While no formal feedback form was distributed, informal verbal feedback suggested increased awareness and ease of use of the template. A follow-up discussion at the end of the second cycle suggested that participants found the standardization helpful, especially when documenting under time pressure during emergency admissions.

Cycle 2

Cycle 2 was a prospective audit of 37 laparoscopic appendectomy cases from September to November 2024. The same proforma was used for data collection. Only procedures performed by general surgery teams were included. Patients undergoing open appendectomy, incidental appendectomy, or those with missing operative notes were excluded. Data extraction was performed independently by two surgical trainees and verified by the audit lead. For each documentation standard, compliance was recorded as a binary variable: documented or not documented. 

Statistical analysis

Data were recorded and analyzed using Microsoft Excel (Microsoft Corporation, Redmond, Washington, United States). Compliance with each of the 34 GIRFT documentation standards was recorded as a binary outcome (documented vs. not documented). Descriptive statistics were used to calculate compliance rates as frequencies and percentages. To compare documentation compliance between Cycle 1 and Cycle 2, the chi-square test was applied for categorical variables. For data with expected cell counts <5, Fisher’s exact test was used. A p-value of <0.05 was considered statistically significant. Cross verification of the statistical output was done using an open-source statistical software (R) to ensure the accuracy of the Chi-square test results.

## Results

**Figure 2:**
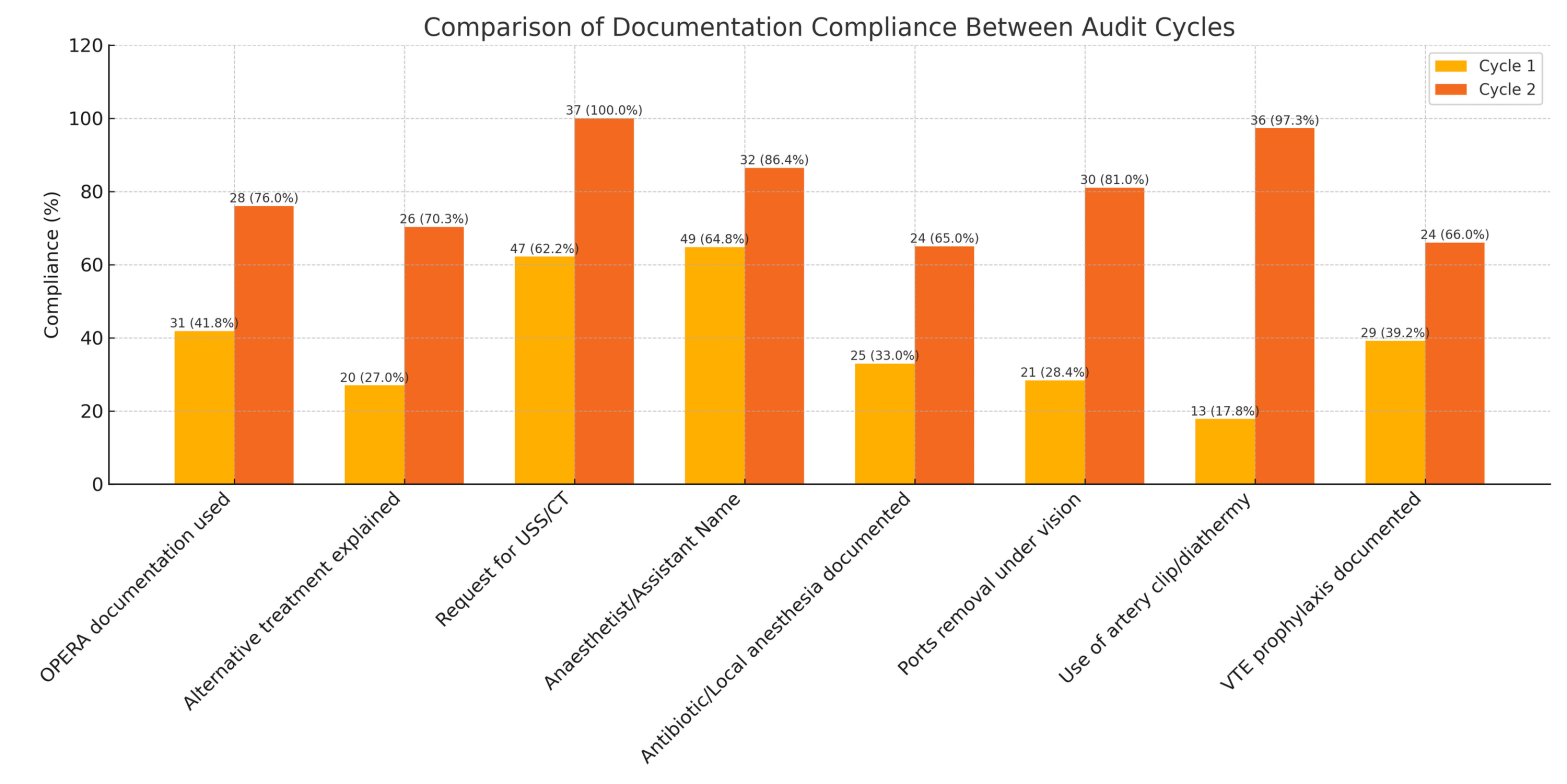
Comparison of selected documentation standards between cycle 1 and cycle 2 OPERA: Operative Electronic Record; USS: ultrasound scan; CT: computed tomography; VTE: venous thromboembolism. Data are presented as the number of compliant cases (n) with percentage in parentheses. Cycle 1: n=75; Cycle 2: n=37.

A total of 112 laparoscopic appendectomy cases were reviewed: 75 in Cycle 1 (May-July 2024) and 37 in Cycle 2 (September-November 2024). Compliance was assessed against 34 documentation standards outlined in the GIRFT recommendations, grouped into preoperative, intraoperative, and postoperative domains (Table 2 and Figure 2).

**Table 2:** Comparison of selected documentation standards between cycle 1 and cycle 2 OPERA: Operative Electronic Record; USS: ultrasound scan; CT: computed tomography; VTE: venous thromboembolism. Data are presented as the number of patients achieving compliance with each standard, shown as n (%).

Documentation Standard	Cycle 1 Compliance (n=75), n (%)	Cycle 2 Compliance (n=37), n (%)
OPERA documentation used	31 (41.8%)	28 (76.0%)
Alternative treatment explained	20 (27.0%)	26 (70.3%)
Request for USS/CT	47 (62.2%)	37 (100.0%)
Anaesthetist/Assistant name documented	49 (64.8%)	32 (86.4%)
Antibiotic/Local anaesthetic use	25 (33.0%)	24 (65.0%)
Ports removed under vision	21 (28.4%)	30 (81.0%)
Use of artery clip/diathermy	13 (17.8%)	36 (97.3%)
Blood loss documented	1 (1.35%)	5 (13.6%)
Drain removal documented	29 (38.9%)	28 (77.0%)
VTE prophylaxis documented	29 (39.2%)	24 (66.0%)
Surgeon’s signature and grade	51 / 12 (67.6% / 16.2%)	32 / 23 (87.0% / 63.0%)
Histopathology result checked	8 (11.0%)	8 (21.6%)
Photography documented	21 (28.4%)	6 (16.2%)

Preoperative documentation

High baseline compliance was noted for key standards such as recording the indication for appendectomy (Cycle 1: 97.2%, Cycle 2: 100%), inflammatory markers (95.9% → 97.3%), and informed consent (100% in both cycles). Significant improvements were seen in documentation of alternative treatment options (27% → 70.3%) and imaging (USS/CT request: 62.2% → 100%). OPERA usage increased from 41.8% to 76%.

Intraoperative documentation

Marked improvements were observed in multiple intraoperative parameters: Anesthetist name: 64.8% → 86.4%, Antibiotic documentation: 33% → 65%, Port insertions under vision: ~62% → 86%, Use of artery clip/diathermy: 17.8% → 97.3%

Postoperative documentation

Postoperative documentation also improved, particularly in: Drain removal documentation: 38.9% → 77%, VTE prophylaxis: 39.2% → 66%, Signature and grade of surgeon: 67.6%/16.2% → 87%/63% Areas requiring further improvement included blood test documentation (2.7% → 13.5%), histopathology follow-up (10.95% → 21.6%), and photography (28.4% → 16.2%).

Several parameters showed statistically significant post-intervention improvement, including documentation of alternative treatment options (p<0.001), use of artery clip/diathermy (p<0.001), and OPERA usage (p=0.002) (Table 3).

**Table 3:** Statistical comparison of documentation compliance between audit cycles Values are expressed as number of compliant cases (n) and percentage (%). Statistical significance determined using chi-square test or Fisher’s exact test as appropriate. A p-value <0.05 was considered statistically significant.

Documentation Standard	Cycle 1 (n=75), n (%)	Cycle 2 (n=37), n (%)	p-value
OPERA documentation used	31 (41.3%)	28 (75.7%)	0.0013
Alternative treatment explained	20 (26.7%)	26 (70.3%)	<0.0001
Request for USS/CT	47 (62.7%)	37 (100.0%)	<0.0001
Anaesthetist/Assistant name documented	49 (65.3%)	32 (86.5%)	0.0333
Antibiotic/Local anaesthetic documented	25 (33.3%)	24 (64.9%)	0.0031
Ports removed under vision	21 (28.0%)	30 (81.1%)	<0.0001
Use of artery clip/diathermy	13 (17.3%)	36 (97.3%)	<0.0001
Blood loss documented	1 (1.3%)	5 (13.5%)	0.0231
Drain removal documented	29 (38.7%)	28 (75.7%)	0.0003
VTE prophylaxis documented	29 (38.7%)	24 (64.9%)	0.0134
Surgeon’s signature documented	51 (68.0%)	32 (86.5%)	0.0481
Surgeon’s grade documented	12 (16.0%)	23 (62.2%)	<0.0001
Histopathology result checked	8 (10.7%)	8 (21.6%)	0.1385
Photography documented	21 (28.0%)	6 (16.2%)	0.2206

## Discussion

This two-cycle prospective audit demonstrates that structured interventions, including implementation of a standardised electronic template and targeted staff education, can significantly improve compliance with documentation standards in laparoscopic appendectomy. In the initial cycle, retrospective data collection revealed suboptimal documentation in several critical areas. The subsequent introduction of a structured OPERA-based template, along with educational reinforcement for both trainees and consultants, contributed to marked improvements in documentation quality. While the documentation template played a significant role in improving compliance, these targeted educational interventions contributed by reinforcing key expectations, increasing familiarity with documentation standards, and highlighting accountability. We believe the combination of repeated exposure and practical demonstrations directly supported the observed improvements in Cycle 2. Our findings align with the broader goals of the GIRFT initiative [3], which emphasises data-driven quality improvement in surgical care through national benchmarking and documentation standardisation.

Operative note audits across the UK and internationally have consistently revealed suboptimal compliance with documentation guidelines. A study from central London found that average compliance increased from 56.1% in the initial audit to 98.2% after implementing a structured proforma, with statistical significance (p < 0.0001) [7]. In contrast, audits in Pakistan showed much lower baseline compliance, with only 47% of laparoscopic appendectomy notes meeting Royal College of Surgeons of England (RCS) standards, and these did not incorporate re‑audit cycles [8]. Such variability underscores the impact of introducing standardised note templates tailored to institutional needs.

The value of dedicated proformas and embedded prompts within EMRs has been reinforced by multiple studies. Torab‑Miandoab et al. reported improvements in legibility, completeness, and speed of documentation after moving to template-led electronic records [9].

Despite our improvements, certain areas, such as documentation of histopathology review and intraoperative photography, remained under-documented. These gaps suggest that passive templates may not suffice; active, system-level prompts or postoperative checklists may be required to achieve sustained adherence. Furthermore, it is important to acknowledge that documentation quality is a surrogate marker. While improved note completeness may enhance team communication and medicolegal assurance, its direct correlation with patient outcomes, such as complication rates or readmissions, remains to be assessed-and represents a valuable direction for future study.

To strengthen sustainability, we recommend integrating documentation standards into onboarding processes, e‑logbooks, and ongoing appraisal frameworks. Beyond internal audit cycles, cross‑site benchmarking through GIRFT or NHS platforms could facilitate broader quality comparisons and motivate continuous improvement. Finally, future work could aim to correlate improvements in documentation with clinical outcomes and retrospective medico‑legal case analysis to validate the clinical significance of these interventions.

This audit reinforces the effectiveness of the GIRFT framework as both a benchmarking and improvement tool. The adoption of EMRs has led to clear advantages, including time efficiency, enhanced data quality, positive professional impact, high user satisfaction, and valuable qualitative feedback [9]. By building on these benefits and actively incorporating user input, healthcare institutions can further refine clinical documentation practices and maximize the effectiveness of EMR systems to support better patient outcomes and care delivery.

Limitations

This audit was conducted at a single district general hospital, which may limit the generalisability of the findings to other settings, particularly tertiary centres or hospitals using different electronic documentation systems. The sample size in Cycle 2 was smaller due to the limited audit window, although the findings still demonstrated statistically and clinically significant improvements. Data were extracted manually from operative notes, which may introduce observer bias despite cross-checking by two independent reviewers. Additionally, reasons for residual non-compliance include user unfamiliarity with the OPERA system, time pressures in emergency settings, and variation in documentation habits among junior staff. Along with this, the audit focused solely on documentation quality and did not assess correlations with patient outcomes, surgical complications, or readmission rates. These areas may warrant future evaluation. 

Recommendations

To sustain and build upon the improvements observed, we recommend continued use of the OPERA-based operative note template across all surgical teams within the Trust. Regular staff education sessions, including the incorporation of documentation standards into induction programmes for new trainees, should be maintained to ensure awareness and compliance. Periodic re-auditing is essential to monitor adherence and identify areas needing further intervention. Additionally, system-level enhancements, such as automated prompts or checklists for frequently omitted fields (e.g., histopathology review, postoperative blood tests), integrating reminders within electronic systems, and incorporating documentation standards into surgical induction or appraisal frameworks may further improve documentation consistency and completeness.

## Conclusions

The introduction of a structured OPERA-based operative note template and targeted staff education led to significant improvements in compliance with GIRFT documentation standards in laparoscopic appendectomy. This audit highlights the effectiveness of low-resource, system-level interventions in standardising surgical documentation and improving quality of care. Continued use of the electronic template, along with periodic re-audits and sustained staff engagement, is recommended to maintain high standards of operative record-keeping.
